# Ethnicity, pre-existing comorbidities, and outcomes of hospitalised patients with COVID-19

**DOI:** 10.12688/wellcomeopenres.16580.2

**Published:** 2021-06-30

**Authors:** Gillian Santorelli, Michael McCooe, Trevor A. Sheldon, John Wright, Tom Lawton

**Affiliations:** 1 Bradford Institute for Health Research, Bradford, West Yorkshire, BD9 6RJ, UK; 2 Institute for Population Health Sciences, Barts and The London NHS Trust, Queen Mary University of London, London, E1 4NS, UK

**Keywords:** COVID-19, ethnicity, mortality, comorbidity

## Abstract

**Background**: The coronavirus disease 2019 (COVID-19) pandemic has resulted in thousands of deaths in the UK. Those with existing comorbidities and minority ethnic groups have been found to be at increased risk of mortality. We wished to determine if there were any differences in intensive care unit (ICU) admission and 30-day hospital mortality in a city with high levels of deprivation and a large community of people of South Asian heritage.

**Methods**: Detailed information on 582 COVID-19-positive inpatients in Bradford and Calderdale between February-August 2020 were extracted from Electronic Health Records. Logistic regression and Cox proportional hazards models were used to explore the relationship between ethnicity with admission to ICU and 30-day mortality, respectively accounting for the effect of demographic and clinical confounders.

**Results**: The sample consisted of 408 (70%) White, 142 (24%) South Asian and 32 (6%) other minority ethnic patients. Ethnic minority patients were younger, more likely to live in deprived areas, and be overweight/obese, have type 2 diabetes, hypertension and asthma compared to white patients, but were less likely to have cancer (South Asian patients only) and COPD. Male and obese patients were more likely to be admitted to ICU, and patients of South Asian ethnicity, older age, and those with cancer were less likely. Being male, older age, deprivation, obesity, and cancer were associated with 30-day mortality. The risk of death in South Asian patients was the same as in white patients HR 1.03 (0.58, 1.82).

**Conclusions**: Despite South Asian patients being less likely to be admitted to ICU and having a higher prevalence of diabetes and obesity, there was no difference in the risk of death compared to white patients. This contrasts with other findings and highlights the value of studies of communities which may have different ethnic, deprivation and clinical risk profiles.

## Introduction

As of the end of 2020, there have been more than two and a half million confirmed cases of coronavirus disease 2019 (COVID-19), resulting in almost 75,000 deaths in the UK alone ^1^. Increasing age and male gender are now well-known risk factors for adverse outcomes from COVID-19 and several studies have demonstrated an increased mortality risk associated with underlying health conditions such as diabetes, obesity and severe respiratory disease ^2^. A large cohort study, using the OpenSAFELY analytics platform has identified that people of Black and South Asian ethnicity have a much higher risk of death associated with COVID-19 than those of white ethnicity that is only partially attributable to co-morbidities, deprivation or other factors ^3^. Additionally, the same study showed a pattern of increasing risk with greater deprivation.

Bradford Teaching Hospitals Foundation Trust (BTHFT) and Calderdale and Huddersfield Foundation Trust (CHFT) are large hospital trusts in West Yorkshire in the UK which consist of three separate acute hospitals and together serve a population of approximately one million people. Bradford is a deprived and ethnically diverse city, with high rates of health problems such as obesity and cardiovascular disease as well as the highest prevalence of diabetes in the UK. Calderdale and Huddersfield, by comparison have a smaller proportion of residents from black, south Asian and minority ethnic backgrounds yet have many areas that suffer poor health outcomes and significant deprivation. The current pandemic has exposed and resulted in inequalities between UK ethnic groups ^4^.

On the basis of early evidence from China and Italy, Bradford teaching hospitals, chose to adopt the widespread early use of enhanced respiratory support in the form of CPAP (Continuous Positive Airway Pressure) and self-proning in the management of more severe COVID-19, with the aims of improving patient outcomes and controlling critical care demand. Preliminary analysis of patients receiving CPAP and mechanical ventilation at BTHFT did not demonstrate an increased mortality rate amongst those of South Asian ethnicity and this observation provided impetus for this multi-centre review of inpatients with a diagnosis of COVID 19 ^5^.

The overall aim of this study was to examine the ethnic, demographic, socio-economic and clinical risk factors associated with outcomes of hospital inpatients who tested positive for COVID-19. The endpoints were ICU admission and 30-day in-hospital mortality.

## Methods

***Sample***: This prospective cohort study includes all patients admitted to Bradford Teaching Hospitals Foundation Trust (BTHFT) between 17/02/2020 – 20/06/2020 and Calderdale and Huddersfield Foundation Trust (CHFT) between 01/03/2020 – 08/08/2020, and who tested positive for severe acute respiratory syndrome coronavirus 2 (SARS-CoV-2) using RT-PCR on admission or during their stay. Bradford has a large South Asian population (24.9%, 20.4% of who are of Pakistani origin; 67.4% white), whereas Calderdale and Kirklees (the Metropolitan Borough in which Huddersfield resides) have a larger white population (89.6% and 79.1% respectively).

****Ethics approval****: The recent Health Service (Control of Patient Information) Regulations 2002 notices requires NHS Trusts and others to process confidential patient information without consent for COVID-19 public health, surveillance and research purposes (see NHS HRA COVID-19 Research guidance), thus no ethical approval or consent for publication was required.

***Data***: Detailed information on ethnicity, demographic characteristics, hospital stay, and pre-existing comorbidities was manually extracted from hospital electronic patient records (EPR) for BTHFT patients; the data for those admitted to CHFT was extracted directly from the data warehouse. Self-defined ethnicity was categorised to reflect the majority ethnic groups in the region: White (British, Irish, any other White), South Asian (Indian, Pakistani, Bangladeshi), and other ethnic minority; patients with missing ethnicity were excluded from the analysis. Potential risk factors for severe COVID-19 were identified *a priori*: age, sex, obesity, and pre-existing comorbidities (diabetes, hypertension, coronary heart disease, asthma, chronic obstructive pulmonary disease (COPD), cancer, chronic renal disease). BMI categories were defined as healthy (<25 kg/m ^2^), overweight (25–29.9 kg/m ^2^) and obese (≥30 kg/m ^2^) based on BMI at hospital admission. Socioeconomic status was estimated using the English Indices of Multiple Deprivation (IMD) quintiles derived from residential postcode. Due to small numbers in the least deprived quintile, IMD 4 and 5 were combined. We excluded patients whose positive test was not within ±14 days of admission.

***Statistical analysis***: Patient characteristics were summarised using number (%) or median (IQR). Differences across the two largest ethnic groups (white and South Asian) were explored using the chi-squared or Fisher’s exact test, and one-way ANOVA or Kruskal-Wallis test. The association between ethnicity and risk factors with admission to ICU was modelled using logistic regression models. Time-to-event analysis was performed to model survival. Time zero was the date of a COVID-19 positive test and patients were censored at discharge from hospital or 30 days from the date of the test. Three Cox proportional hazards models were used to assess the effect of ethnicity on survival: model 1 was unadjusted, model 2 was adjusted for age and sex, and model 3 additionally adjusted for South Asian ethnicity (reference: White), age on admission (reference: 50–59 years), IMD and pre-existing comorbidities (obesity, type 2 diabetes, hypertension, cardiovascular disease, asthma, chronic obstructive pulmonary disease, cancer and renal disease). Due to small numbers, models were not fitted for type 1 diabetes, though it was included in the comorbidity count. All models used robust variance estimates to account for intragroup correlation within hospital trust. Effect measures are presented as odds ratios (OR) or hazard ratios (HR) with 95% confidence intervals. Due to heterogeneity and small numbers, patients from other ethnic minorities were not included in the logistic regression or survival analyses. To assess the effect of missing data, a sensitivity analysis of the multivariable Cox model was conducted using 50 sets of multiple imputed datasets using chained equations. All analyses were conducted using STATA/SE software (Stata/SE 15, StataCorp, College Station, TX, USA), and plots were constructed using the *forestplot* package (version 1.10.1) in R version 3.6.1 ^6^.

## Results

***Sample***: Information was extracted for 737 patients. Those with missing ethnicity (n=48), comorbidity (n=78), and deprivation data (n=19) or aged <18 years (n=10) were excluded from the analysis, leaving a final sample of 582: 408 (70.1%) white; 142 (24.4%) South Asian and 32 (5.5%) from other ethnic groups.

Table 1^7^ presents the socio-demographic and clinical characteristics of the sample. Patients, particularly of those of other ethnic minorities, were more likely to be male. Ethnic minority groups were significantly younger compared to white patients and were more likely to live in deprived areas. White patients had a lower prevalence of obesity, type 2 diabetes, hypertension, and asthma compared to ethnic minority patients, but a higher prevalence of COPD. South Asian patients had a lower prevalence of cancer compared to white and other ethnic minority patients but had a higher number of comorbidities. Most of the sample had at least one comorbidity, with half having three or more. Most (96%) patients had their first positive test or formal diagnosis either before or within 7 days of admission and were therefore community-acquired. The median length of hospital stay was 6 (IQR 3–12) and did not differ between white and South Asian ethnic groups.

**Table 1.** Characteristics of COVID-19 positive inpatients. Values are n (%) or median (IQR).

	All	White	South Asian	Other ^1^	P-value ^2^
	N=582	N=408 (70.1%)	N=142 (24.4%)	N=32 (5.5%)	
***Socio-demographic characteristics***					
Male	329 (56.5)	227 (55.6)	77 (54.2)	25 (78.1)	0.771
Age on admission (years)					<0.001
18 – 49	91 (15.6)	34 (8.3)	49 (34.5)	8 (25.0)	
50 – 59	84 (14.4)	49 (12.0)	29 (20.4)	6 (18.8)	
60 – 69	113 (19.4)	75 (18.4)	29 (20.4)	9 (28.1)	
70 – 79	105 (18.0)	92 (22.6)	11 (7.8)	2 (6.3)	
80+	189 (32.5)	158 (38.7)	24 (16.9)	7 (21.9)	
IMD					<0.001
1 (most deprived)	264 (45.4)	139 (34.1)	102 (71.8)	23 (71.9)	
2	131 (22.5)	94 (23.0)	31 (21.8)	6 (18.8)	
3	84 (14.4)	80 (19.6)	3 (2.1)	1 (3.1)	
4/5 (least deprived)	103 (17.7)	95 (23.3)	6 (4.2)	2 (3.3)	
Trust					<0.001
BTHFT	249 (42.8)	135 (33.1)	96 (67.6)	18 (56.3)	
CHFT	333 (57.2)	273 (66.9)	46 (32.4)	14 (43.8)	
***Clinical characteristics***					
BMI (kg/m ^2^)					
Healthy weight (<25)	280 (48.1)	217 (53.2)	50 (35.2)	13 (40.6)	0.001
Overweight (25-29.9)	148 (25.4)	95 (23.3)	42 (29.6)	11 (34.4)	
Obese (30+)	154 (26.5)	96 (23.5)	50 (35.2)	8 (25.0)	
Diabetes type 1	10 (1.7)	6 (1.5)	1 (0.7)	3 (9.4)	0.483
Diabetes type 2	208 (35.6)	119 (29.2)	75 (52.8)	13 (40.6)	<0.001
Hypertension	270 (46.4)	175 (42.9)	77 (54.2)	18 (56.3)	0.020
Cardiovascular disease	176 (30.2)	130 (31.9)	38 (26.8)	8 (25.0)	0.256
Asthma	91 (15.6)	52 (12.8)	29 (20.4)	10 (31.3)	0.026
COPD	78 (13.4)	67 (16.4)	10 (7.0)	1 (3.1)	0.006
Cancer	47 (8.1)	39 (9.6)	4 (2.8)	4 (12.5)	0.010
Renal disease	146 (25.1)	105 (25.7)	32 (22.5)	9 (28.1)	0.448
Comorbidities					0.916
0	64 (11.0)	45 (11.0)	15 (10.6)	4 (12.5)	
1-2	215 (36.9)	150 (36.8)	55 (38.7)	10 (31.3)	
3+	303 (52.1)	213 (52.2)	72 (50.7)	18 (56.3)	
***Admission details***					
Covid-19 positive on/within 7 days of admission	560 (96.2)	389 (95.3)	140 (98.6)	31 (96.9)	0.082
Length of hospital stay (days) ^3^	6 (3–12)	7 (3–12)	5 (2–9)	8 (3–13.5)	0.002
Admitted to ICU on arrival	30 (5.2)	20 (4.9)	10 (7.0)	0	0.333
Admitted to ICU at any time during hospital stay	57 (9.8)	37 (9.1)	17 (12.0)	3 (9.4)	0.317
Length of ICU stay (days)	10 (6-23)	8 (6–15)	7 (5v40)	21 (12–25)	0.834
***Outcomes***					
Died in hospital	200 (34.4)	164 (40.2)	29 (20.4)	7 (21.9)	<0.001
Died in hospital within 30 days of positive COVID-19 test	189 (32.5)	154 (37.8)	28 (19.7)	7 (21.9)	<0.001

^1^Black=18, Other Asian=2, Mixed/other=15. ^2^ P-value for difference between white and South Asian ethnic groups. ^3^ 14 patients not discharged at time of data extraction. IMD=Index of Multiple Deprivation; BTHFT=Bradford Teaching Hospitals Foundation Trust; CHFT=Calderdale and Huddersfield Foundation Trust; BMI=body mass index; COPD=chronic obstructive pulmonary disease; ICU=intensive care unit.

**Figure 1.**
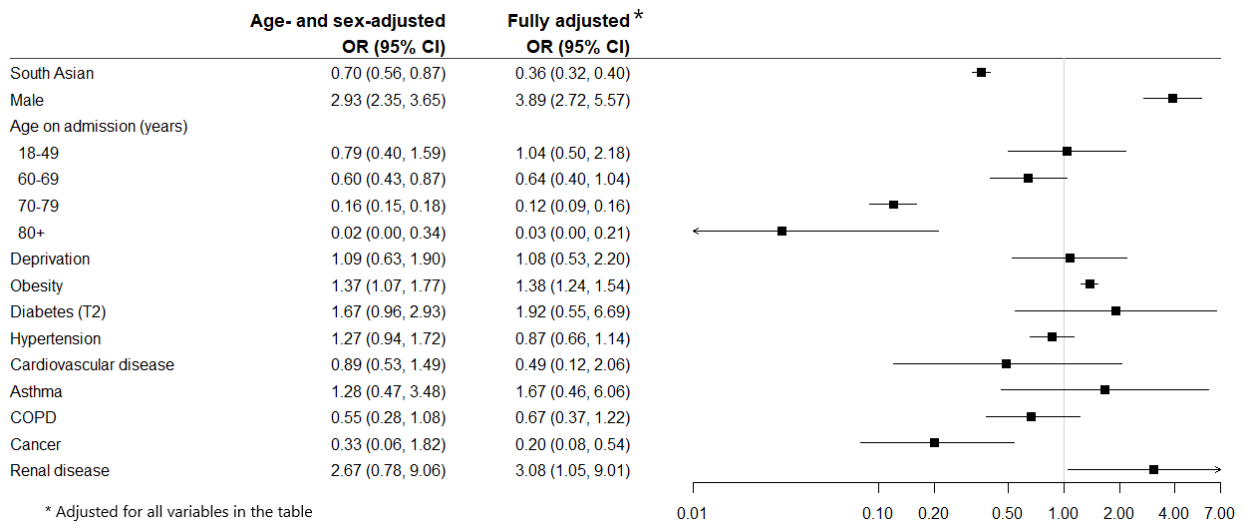
Odds Ratios (95% CI) for risk of ICU admission at any time during inpatient stay.

***ICU admission***: There were no ethnic differences in the proportion of patient’s admitted to ICU on arrival or during their inpatient stay. Figure 1 presents the odds ratios for age- and sex-adjusted and fully adjusted logistic regression models for predictors of ICU admission. In the fully adjusted model, males, and patients with obesity and renal disease were more likely to be admitted whereas older patients, the South Asian ethnic group and patients with cancer were less likely. The median length of stay in ICU was 10 (IQR 6–22), and although slightly higher in South Asian patients it was not statistically significantly different to that of white patients.

**Figure 2.**
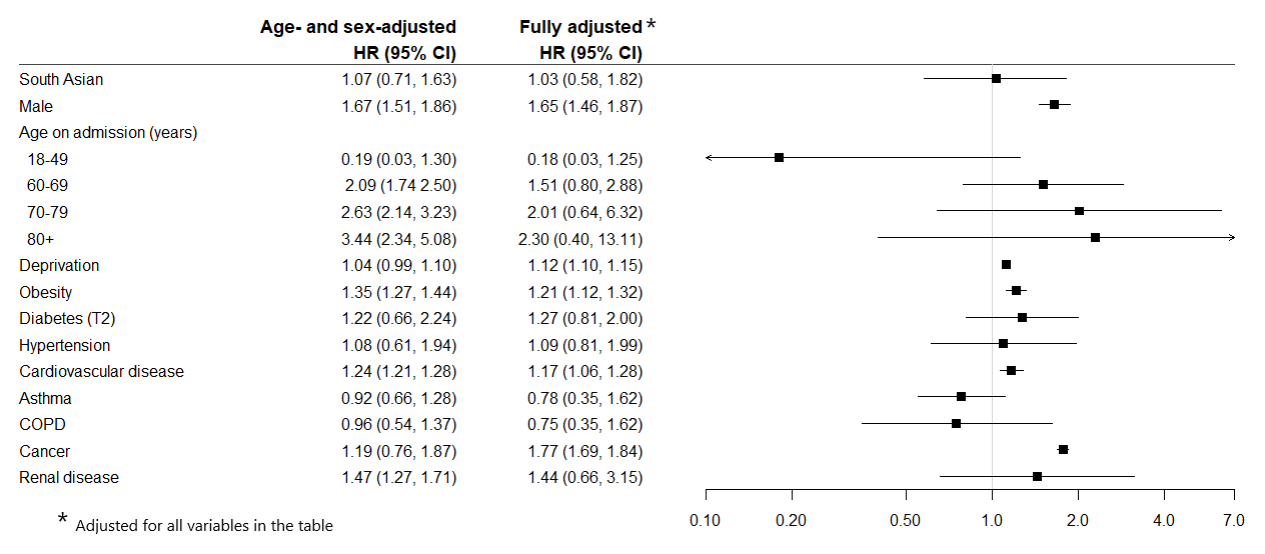
Hazard Ratios (95% CI) for risk of 30 day in-hospital mortality following a positive COVID-19 test.

***30 day in-hospital mortality***: The HRs for ethnic group, socio-demographic and clinical characteristics in the age- and sex-adjusted and fully adjusted models are presented in Figure 2. The unadjusted risk of death was lower in South Asian compared to white patients: HR 0.65 (0.60, 0.72). However, after adjusting for age and sex there was no indication of a higher or lower risk in-hospital death in South Asian patients within 30 days of testing positive for COVID-19 compared to white patients: HR 1.07 (0.71, 1.63) or the fully adjusted model: HR 1.03 (0.58, 1.82). Risk of dying within 30 days was higher in males, older people, increasing deprivation, and those with obesity, cardiovascular disease and cancer. The risk of death was double in those with multimorbidity (three or more conditions) compared to those with no comorbidities: age- and sex-adjusted HR 2.00 (1.88, 2.13). When stratified by ethnic group, the risk in patients with three or more comorbidities was higher in South Asian compared to white patients: HR 2.96 (2.33, 3.74) vs 1.73 (1.58, 1.89) respectively. The sensitivity analysis revealed a similar risk of 30-day mortality in South Asian patients as the complete cases analysis (HR 1.00 (0.72, 1.40)), see Table 2^7^.

**Table 2.** Fully adjusted sensitivity analysis of risk of 30 day in-hospital mortality using imputed data.

	Fully adjusted HR (95% CI)
South Asian	1.00 (0.72, 1.40)
Male	1.61 (1.12, 2.33)
Age on admission (years)	
18 – 49	0.11 (0.01, 0.96)
50 – 59	1.00
60 – 69	1.85 (1.00, 3.44)
70 – 79	2.50 (1.16, 5.37)
80+	3.07 (1.86, 5.04)
Deprivation	1.01 (0.94, 1.08)
Obesity	1.23 0.88, 1.73)
Diabetes (T2)	1.05 (0.65, 1.71)
Hypertension	0.91 (0.86, 0.96)
Cardiovascular disease	1.22 (1.08, 1.39)
Asthma	0.96 (0.57, 1.62)
COPD	0.84 (0.64, 1.10)
Cancer	1.08 (0.52, 2.26)
Renal disease	1.24 (0.78, 1.97)

## Discussion

We can view the progression of a deteriorating patient with COVID-19 along a pathway: they must become infected, symptomatic, and then deteriorate and be appropriate for hospital treatment. To receive ICU treatment, they must be normally well enough that it is felt they will benefit, but also acutely unwell enough that it is necessary. It is clear that in Bradford at least, patients who were not felt appropriate for ICU represented the vast majority of deaths ^5^. Analyses of mortality risk in COVID-19 must therefore pay attention to the point at which the patient becomes at-risk. In this study, as with ISARIC ^8, 9^, the patient has already caught the disease and deteriorated to the point of hospital admission, and hospital admission has been deemed appropriate. By comparison QCOVID and OpenSAFELY incorporate the risk of contracting the disease, becoming symptomatic, and deteriorating to the point of hospital admission. With a wide variety of infection rates across the UK ^1^, the risks of contracting COVID-19 become an important part of the model.

This study agrees with other work that the risk of hospital mortality rises with increasing age and male sex, as well as obesity, diabetes, and a variety of other comorbidities. The same factors seem to relate to a risk of ICU admission, except that there is a more complex relationship in that people with some comorbidities (particularly cancer) or higher age may be considered too frail to benefit from intensive treatment despite severe disease.

However, we found that South Asian ethnicity was associated with a reduced risk of admission to ICU and did not find evidence of an increased risk of death. This contrasts with the larger ISARIC study which found that South Asian ethnicity was associated with both ICU admission and mortality. We also found that deprivation appeared to predict mortality, though its relation to ICU admission was more uncertain.

Clearly this study cannot on its own explain why results appear to differ from a national picture; though it is important to recognise that in studies with a broader geographical footprint there is something lost by a simplistic approach to ethnicity. South Asian people in the area represented in this study are overwhelmingly from Pakistan, and far more likely to live in a deprived area than white patients ^10^. This may therefore represent a genuine difference in risk compared with what is likely to be a relatively heterogeneous “South Asian” group in work such as ISARIC. Bradford particularly has many large, multigenerational households, which may be protective for severe COVID-19 despite the high prevalence of other risk factors such as obesity and diabetes ^2^.

Treatment in this cohort’s hospitals may not be representative of treatment received across the UK. Particularly, Bradford Royal Infirmary used early CPAP at a rate roughly twice the national average ^5^. It is possible that this may have had a differential effect on mortality in the younger South Asian population. Contextual factors such as close engagement with the South Asian community by the local Scientific Advisory Group and District Gold Command may have helped to counter misinformation and prevent late presentation to hospital, which may be a cause of increased risk in some communities ^11, 12^.

Lastly, and most worryingly as an option, the prevalence of misinformation about COVID-19 in Bradford’s South Asian community may have made people scared to attend hospital and resulted in more dying at home ^13^; though it would be expected that this would lead to an under-representation of the milder cases rather than the more severe ones that would explain the associations seen. In short, it is likely that “South Asian” as an ethnicity in the areas studied reflects a different profile to those in other studies.

Comprising two centres, this study is relatively small compared to work such as ISARIC, but it has the advantage of covering a relatively local population where it is more reasonable to model people as grouped by ethnicity. Only the first wave of COVID-19 infections is represented, but anecdotally the second wave is quite different in character so it may be best analysed separately. The data has been collected specifically for this project and as a result is fairly complete; and adjustment has been made for all the main potential confounders identified in other work.

This study has its limitations. We acknowledge that our sample size was small. The difference in the proportion of white and South Asian patients admitted to ICU was small (δ=0.03) and thus we had low power to draw robust conclusions for this outcome. However, the difference in 30-day hospital mortality between the ethnic groups was large enough to achieve a power of 0.99. The time-periods of the data available from the two hospitals differ slightly, but it is unlikely that this would have impacted on the outcomes. Finally, we were not able to identify whether the patients in our sample included clinicians or health workers, but there is no reason to suspect that outcomes would be different depending on occupation.

We would conclude that there is no evidence for people of South Asian ethnicity hospitalised with COVID-19 having a worse outcome. Findings in large studies may not be replicated in individual local areas, due to the complex interplay between ethnicity, deprivation, living conditions, and comorbidities. Particularly during the pandemic, it is vital to provide local evidence to local communities in order to reassure about risk and communicate the importance of seeking health service attention where necessary ^14^.

## Data availability

### Underlying data

Harvard Dataverse: Replication Data for: Ethnicity, pre-existing comorbidities, and outcomes of hospitalised patients with COVID-19. https://doi.org/10.7910/DVN/RRCQEO ^7^

This project contains the following underlying data:

- DatasetForPublication.xls

Data are available under the terms of the Creative Commons Zero "No rights reserved" data waiver (CC0 1.0 Public domain dedication).
