# Supplementary material for: Ethnicity, pre-existing comorbidities, and outcomes of hospitalised patients with COVID-19
Source: Wellcome Open Res. 2021 Jun 30;6:32. Originally published 2021 Feb 16. [Version 2] doi: 10.12688/wellcomeopenres.16580.2 (PMC8408538; doi:10.12688/wellcomeopenres.16580.2)
Supplement: Peer review history

# Peer review: Reviewer response for version 2

**DOI:** 10.21956/wellcomeopenres.18784.r45371

**Published:** 2021-08-31

**Authors:** Olufemi A. Erinoso

**Affiliations:** 1 Department of Oral and Maxillofacial Surgery, Lagos State University Teaching Hospital, Ikeja, Nigeria

**General Comments:**

 Thank you for the opportunity to review this manuscript which addresses an important subject. This is an interesting manuscript about ethnicity, pre-existing co-morbidities, and COVID-19 outcome in hospitalized patients. The findings validate and add to the existing literature on COVID-19 risk factors, and can help policymakers and researchers understand policy options and areas for additional study. However, I have some minor comments, which are stated below.

**Specific comments**:

- Methods section: Ethnicity, as used in this paper, represents a sociopolitical construct, and can help address health disparities. However, aggregating ethnicities runs the risk of limiting the usefulness of ethnicity as a representation of lived experience, and this might in turn yield misleading results. For example, some Irish (Northern Ireland) are British, while others are Irish (Ireland). Similarly, the South-Asian categories- Indian, Pakistan, and Bangladesh- have distinct ethnicities in each of these countries, especially India.
- Can the authors clarify how they arrived at this distinction and what theory or rationale guided their use of these categories? This can be a paragraph in the methodology section and described under the subsection ‘Data’.
- Methods section: Another concern with the paper is the use of ‘ethnicity' as a mutually exclusive category in analyses, without accounting for multiple identities (for example- a ‘Pakistani’ Father and ‘White’ Mother). Does the Bradford Teaching Hospitals Foundation Trust (BTHFT) electronic patient record system allow patients to identify with ‘multiple identities’? If not, this can be mentioned under the study limitations, or if there is an explanation for this, it can be stated in the methods section.
- Results section, Table 1: The ‘Other’ category based on the footnote is n=35 [1Black=18, Other Asian=2, Mixed/other=15.], but the table states N=32 (5.5%). Please clarify or correct.
- Discussion section: Can the authors provide a reference(s) to the ‘other work’ referred to here: “This study agrees with other work that the risk of hospital mortality rises with increasing age and male sex, as well as obesity, diabetes, and a variety of other co-morbidities.”
- Discussion section: Is it possible that the difference seen with the south Asian population in the current study compared with Harrison *et al.* study (ISARIC) is due to the lower rates of some co-morbidities (cardiovascular disease and COPD) in the south Asian population compared to Whites?
- Also, could smoking rates between the white and south Asian populations be a potential confounder? This could have been included in the models. The authors can also state this as a limitation if data on smoking history was unavailable.
- Discussion section: I suggest the authors include the citation for the larger ISARIC study referred to here: “Concerning the statement “This contrasts with the larger ISARIC study, which found that south Asian ethnicity was associated with both ICU admission and mortality.”

**Reference**:

 Reference 5 has now been peer-reviewed and published. I suggest the citation be updated: Lawton T, Wilkinson K, Corp A, et al. Reduced critical care demand with early CPAP and proning in COVID-19 at Bradford: A single-centre cohort. Journal of the Intensive Care Society. May 2021. doi:10.1177/17511437211018615

Is the work clearly and accurately presented and does it cite the current literature?

Yes

If applicable, is the statistical analysis and its interpretation appropriate?

Yes

Are all the source data underlying the results available to ensure full reproducibility?

Yes

Is the study design appropriate and is the work technically sound?

Partly

Are the conclusions drawn adequately supported by the results?

Yes

Are sufficient details of methods and analysis provided to allow replication by others?

Yes

Reviewer Expertise:

Public Health

I confirm that I have read this submission and believe that I have an appropriate level of expertise to confirm that it is of an acceptable scientific standard.

## References

[1] : Ethnicity, pre-existing comorbidities, and outcomes of hospitalised patients with COVID-19. *Wellcome Open Research*.2021;6: 10.12688/wellcomeopenres.16580.210.12688/wellcomeopenres.16580.2PMC840853834522788

[2] : Reduced critical care demand with early CPAP and proning in COVID-19 at Bradford: A single-centre cohort. *Journal of the Intensive Care Society*.2021; 10.1177/1751143721101861510.1177/17511437211018615PMC967991036751359

# Peer review: Reviewer response for version 2

**DOI:** 10.21956/wellcomeopenres.18784.r44701

**Published:** 2021-08-02

**Authors:** Janice Atkins

**Affiliations:** 1 Epidemiology and Public Health Group, University of Exeter Medical School, Exeter, UK

The authors have responded adequately to all of the reviewer comments.

Is the work clearly and accurately presented and does it cite the current literature?

Yes

If applicable, is the statistical analysis and its interpretation appropriate?

Yes

Are all the source data underlying the results available to ensure full reproducibility?

Yes

Is the study design appropriate and is the work technically sound?

Partly

Are the conclusions drawn adequately supported by the results?

Yes

Are sufficient details of methods and analysis provided to allow replication by others?

Yes

Reviewer Expertise:

Epidemiology.

I confirm that I have read this submission and believe that I have an appropriate level of expertise to confirm that it is of an acceptable scientific standard.

# Peer review: Reviewer response for version 1

**DOI:** 10.21956/wellcomeopenres.18272.r42661

**Published:** 2021-03-05

**Authors:** Janice Atkins

**Affiliations:** 1 Epidemiology and Public Health Group, University of Exeter Medical School, Exeter, UK

This prospective cohort study explored the associations between ethnicity, demographics and comorbidities with outcomes of hospital inpatients who tested positive for COVID-19 (endpoints were admission to ICU and 30-day mortality).

 Major comments:

- The sample size was quite small, so is there adequate power to draw valid conclusions?
- It would be interesting to know who was admitted for COVID-19 and who acquired it during their stay in hospital.
- Did the sample include clinicians/health care workers, or were these excluded?

Minor comments:

- Abstract – The abstract says the sample consisted of 622 COVID-19 positive inpatients but the Results section is inconsistent and says 582 were included.
- Methods: Sample - Why do time periods studied for the two different hospitals differ? It would be more consistent to include the same time periods.
- Methods: Statistical analysis - “Three Cox proportional hazards models were used to assess the effect ethnicity on survival”. There seems to be a typo – should this be “effect **of** ethnicity”?
- Figures 1, Figure 2 and Table 2 – it would be useful to add a foot note for what was included in “Fully Adjusted” models.

Is the work clearly and accurately presented and does it cite the current literature?

Yes

If applicable, is the statistical analysis and its interpretation appropriate?

Yes

Are all the source data underlying the results available to ensure full reproducibility?

Yes

Is the study design appropriate and is the work technically sound?

Partly

Are the conclusions drawn adequately supported by the results?

Yes

Are sufficient details of methods and analysis provided to allow replication by others?

Yes

Reviewer Expertise:

Epidemiology.

I confirm that I have read this submission and believe that I have an appropriate level of expertise to state that I do not consider it to be of an acceptable scientific standard, for reasons outlined above.

# Response

**Published:** 2021-05-26

**Authors:** Gillian Santorelli

**Affiliations:** Bradford Hospitals National Health Service Trust, UK

We thank the reviewer for taking the time to read our article. We will amend the article in response to your comments, but in the meantime, here are our responses:

**The sample size was quite small, so is there adequate power to draw valid conclusions?**

 We acknowledged our sample size is small in the manuscript. The difference in the proportion of white and South Asian patients admitted to ICU was very small (δ=0.03), and thus the power is low. However, the large difference in the proportion of patients in the two ethnic group was large enough to achieve a power of 0.99. Further, most of our findings agree with larger studies.

**It would be interesting to know who was admitted for COVID-19 and who acquired it during their hospital stay.**

 The official definition is that the virus is (1) community acquired if there was a first positive test or formal diagnosis before admission, or up to 7 days (inclusive) after admission, (2) probably hospital acquired if the first positive test or formal diagnosis was 8-14 days after admission, and (3) definitely hospital acquired if the first positive test or formal diagnosis was 15+ days after admission. We restricted our analysis to those who had a positive test +/- 14 days of admission, and using the above definition found that 560 patients acquired the virus in the community, and 22 were probably hospital acquired.

**Did the sample include clinicians/health care workers or were these excluded?**

 We only included people who were admitted to the hospital. Some of these may have been healthcare workers but due to anonymisation of the sample we were not able to identify these. However, all patients were treated the same and there is no reason to suspect that outcomes would be different depending on occupation.

**The abstract says the sample consisted of 622 COVID-19 positive inpatients but the Results section is inconsistent and says 582 were included.**

 This is an error on our part and will be amended in version 2.

**Why do time periods studied for the two hospital differ? It would be more consistent to include the same time periods.**

 We utilised all the data that was available to us. As already mentioned, the sample size was already relatively small, and we wished to include as many patients as we could. It is unlikely that the different time periods would have impacted on the outcomes.

**Statistical analysis – “Three Cox proportional hazards models were used to assess the effect ethnicity on survival”. There seems to be a type – should this be “effect of ethnicity”.**

 We thank the reviewer for bringing this to our attention. We will amend this in version 2.

**Figures 1, Figure 2 and Table 2 – it would be useful to add a foot note for what was included in “Fully adjusted” models.**

 We will include this information in version 2.
